# Expression of Glial-Cell-Line-Derived Neurotrophic Factor Family Ligands in Human Intervertebral Discs

**DOI:** 10.3390/ijms242115874

**Published:** 2023-11-01

**Authors:** Tatsuya Iwasaki, Koji Akeda, Koki Kawaguchi, Junichi Yamada, Takahiro Hasegawa, Norihiko Takegami, Tatsuhiko Fujiwara, Akihiro Sudo

**Affiliations:** Department of Orthopedic Surgery, Mie University Graduate School of Medicine, 2-174 Edobashi, Tsu City 514-8507, Mie, Japan; tatsuyaiwasaki0710@gmail.com (T.I.); k-kawaguchi@med.mie-u.ac.jp (K.K.); yamada-j@med.mie-u.ac.jp (J.Y.); hasegawa-t@med.mie-u.ac.jp (T.H.); n-takegami@med.mie-u.ac.jp (N.T.); tatsuhiko-f@med.mie-u.ac.jp (T.F.); a-sudou@med.mie-u.ac.jp (A.S.)

**Keywords:** intervertebral disc degeneration, glial-cell-line-derived neurotrophic factor family ligands, human

## Abstract

Glial-cell-line-derived neurotrophic factor (GDNF) family ligands (GFLs) contribute to the sensitization of primary afferents and are involved in the pathogenesis of inflammatory pain. The purpose of this preliminary study was to examine the expression of other GFLs (neurturin (NRTN), artemin (ARTN), persephin (PSPN)) and receptors in human IVD cells and tissues exhibiting early and advanced stages of degeneration. Human IVD cells were cultured as a monolayer after isolation from the nucleus pulposus (NP) and anulus fibrosus (AF) tissues. The mRNA expression of NRTN, ARTN, PSPN, and their receptors (GFRA2–GFRA4) was quantified using real-time PCR. Protein expression was evaluated using immunohistochemistry and Western blotting. The expression of NRTN, ARTN, PSPN, and their co-receptors (GFRA2-GFRA4) was identified in human IVD cells at both mRNA and protein levels. A trend was noted wherein the mRNA expression of ARTN, PSPN, and GFRA2 was upregulated by IL-1β treatment in a dose-dependent manner. The percentages of immunopositive cells in the advanced degenerate stage of ARTN, PSPN, and GFRA2 were significantly higher than those in the early degenerate stage. Their expression was enhanced in advanced tissue degeneration, which suggests that GFLs (ARTN and PSPN) may be involved in the pathogenesis of discogenic pain.

## 1. Introduction

Low back pain (LBP) is one of the most common health concerns that affect activities of daily living and the quality of life [1]. The global point prevalence of LBP reported in a systematic review including a total of 165 general population studies from 53 countries was reported to be 11.9% and the prevalence at 1 month was 23.2% [2]. In the United States, the total medical costs of LBP exceed USD 100 billion per year [3]. Multifactorial causes and anatomical features of the lumbar spine contribute to the pathogenesis of chronic LBP [1]. Recent epidemiological studies in large population samples [4,5,6] and cross-sectional clinical studies have provided evidence that intervertebral disc (IVD) degeneration is one of the main causes of LBP.

IVDs, lying between adjacent vertebrae, consist of interwoven fibrous layers of tissues in the annulus fibrosus (AF), which surrounds an inner gelatinous tissue and the nucleus pulposus (NP). IVD degeneration is characterized by changes in both the cellular and extracellular matrix and gradually leads to structural breakdown and impaired IVD function [7]. Normal IVDs are basically avascular and are non-neural tissues; however, sensory and post-ganglionic sympathetic nerve fibers originating from the sinuvertebral nerve are only present in the outer layer of the AF under normal conditions [8,9].

**Figure 1:**
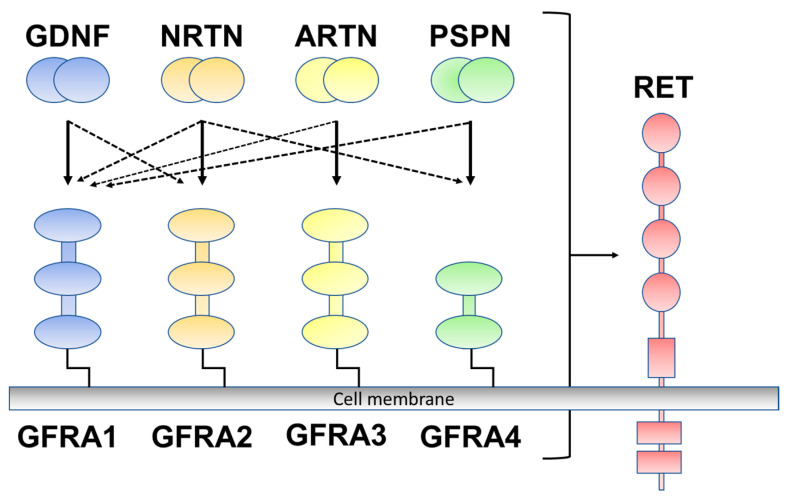
Glial-cell-line-derived neurotrophic factor (GDNF) family ligands and GDNF family receptor alpha 1 to alpha 4 (GFRA1 to A4) are rearranged during transfection (RET). GDNF preferentially binds to GFRA1, neurturin (NRTN) to GFRA2, artemin (ARTN) to GFRA3, and persephin (PSPN) to GFRA4 (solid line). Crosstalk occurs between ligand and co-receptors (dotted line).

Glial-cell-line-derived neurotrophic factor (GDNF) family ligands (GFLs), belonging to the transforming growth factor (TGF)-β superfamily, include GDNF, neurturin (NRTN), artemin (ARTN), and persephin (PSPN). GFLs signal through glycosylphosphatidylinositol (GPI)-anchored co-receptors (GDNF family receptor alpha 1 to alpha 4 (GFRA1–GFRA4)) as the binding component of the ligand and are rearranged by transfection (RET) receptor tyrosine kinase as a signaling component [10]. GDNF preferentially binds to one of the cognate co-receptors (GFRA1), NRTN to GFRA2, ARTN to GFRA3, and PSPN to GFRA4 (Figure 1) [11]. GFLs have been well described to exert growth and maintenance functions in the peripheral and central nervous systems; however, GFLs are also expressed outside of the nervous system [12]. Previous studies have reported that GFLs are expressed in several human tissues in association with immune cells, including the skin, thymus, lung, and spleen, and their expression is upregulated in inflammatory environments [12,13]. There is also accumulating evidence that GFL signaling is involved in the pathogenesis of inflammatory pain [12]. GFLs have been reported to contribute to the sensitization of primary afferents of the skin [14,15], muscle, and bone tissues [16,17].

We have previously reported that GDNF and its associated co-receptors (GFRA1 and RET) are expressed in human IVDs [18]. Furthermore, the percentage of immunopositive GDNF cells was significantly increased in human IVD tissues with the advanced stage of degeneration compared to those in the early stages. Therefore, we suggested the possibility that the increased expression of GDNF within degenerated IVDs may be associated with the pathogenesis of discogenic pain. However, at present, whether the other GFLs, including NRTN, ARTN, and PSPN and their co-receptors (GFRA2 to GFRA4), are expressed in human IVDs has not been investigated.

The purpose of this study was (1) to examine the expression of NRTN, ARTN, and PSPN and their co-receptors (GFRA2 to GFRA4), (2) to evaluate the effects of a pro-inflammatory cytokine on their expression in human IVD cells, and (3) to immunohistochemically examine their expressions in human IVD tissues with different grades of degeneration.

## 2. Results

### 2.1. Detection of mRNA Expression of the GFL Genes in Human IVD Cells

The expression of NRTN, ARTN, PSPN, GFRA2, GFRA3, and GFRA4, quantified using real time-PCR, and mRNA expression were clearly identified in human AF and NP cells. There were no significant differences in the mRNA expression of NRTN, ARTN, PSPN, GFRA2, GFRA3, and GFRA4 between the AF and NP cells (relative expression (AF vs. NP): NRTN, 1.30 ± 1.13 vs. 0.65 ± 0.07; ARTN, 0.43 ± 0.79 vs. 0.11 ± 0.09; PSPN, 0.35 ± 0.37 vs. 0.98 ± 1.60; GFRA2, 0.18 ± 0.21 vs. 0.10 ± 0.11; GFRA3, 1.98 ± 2.54 vs. 1.03 ± 0.05; and GFRA4, 13.8 ± 14.6 vs. 2.11 ± 2.64).

### 2.2. Effect of IL-1β Stimulation on Gene Expression

**Figure 2:**
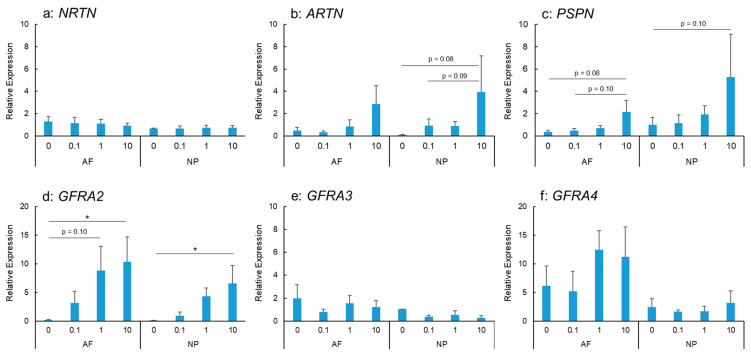
Effects of interleukin-1 beta (IL-1β) on the mRNA expression of neurturin (NRTN) (**a**), artemin (ARTN) (**b**), persephin (PSPN) (**c**), GDNF family receptor alpha 2 (GFRA2) (**d**), GFRA3 (**e**), and GFRA4 (**f**) in human anulus fibrosus (AF) and nucleus pulposus (NP) cells. * *p* < 0.05, one-way analysis of variance followed by post hoc analysis (Fisher’s least significant difference). Error bars: standard error (S.E.).

IL-1β stimulation did not show any significant changes in NRTN mRNA expression in AF and NP cells (Figure 2a). ARTN mRNA expression was stimulated by IL-1β treatment in a dose-dependent manner in the AF and NP cells (Figure 2b); however, this did not reach statistical significance. PSPN mRNA expression in the AF and NP cells tended to be upregulated by IL-1β stimulation in a dose-dependent manner (Figure 2c).

IL-1β stimulation upregulated the mRNA expression of GFRA2 in the AF and NP cells in a significant and dose-dependent manner (Figure 2d). The expression was significantly higher in IL-1β-treated cells (IL-1β: 10 ng/mL) than in the controls for both the AF and NP cells (*p* < 0.05 for both). However, the mRNA expression of GFRA3 did not differ significantly between the control and IL-1β-treated cells (IL-1β: 0.1, 1.0, and 10 ng/mL) for both the AF and NP cells (Figure 2e). GFRA4 mRNA expression in the AF and NP cells was not significantly stimulated by IL-1β treatment (Figure 2f).

### 2.3. Protein Expression using Western Blotting Analyses

**Figure 3:**
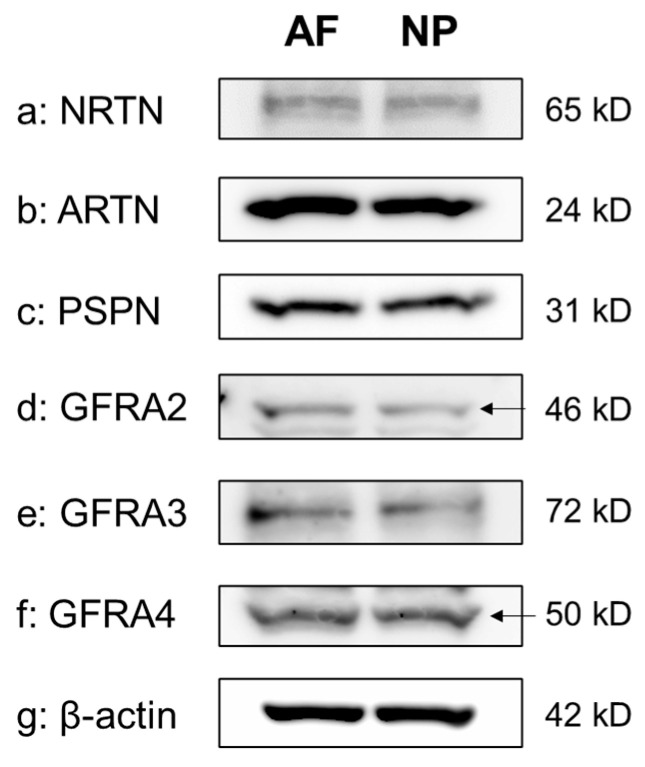
Western blotting showing neurturin (NRTN) (**a**), artemin (ARTN) (**b**), persephin (PSPN) (**c**), GDNF family receptor alpha 2 (GFRA2) (**d**), GFRA3 (**e**), and GFRA4 (**f**) expression in cultured human anulus fibrosus (AF) and nucleus pulposus (NP) cells. β-actin (**g**) served as a loading control. Arrow indicates protein band corresponding to the molecular weight.

The Western blot analyses of cell lysates clearly showed the presence of a single protein band for NRTN (65 kDa), ARTN (24 kDa), PSPN (31 kDa), GFRA2 (46 kDa), GFRA3 (72 kDa), and GFRA4 (50 kDa) in both the NP and the AF cells. β-actin expression was similar between AF and NP cells (Figure 3).

### 2.4. Immunohistochemical Analyses

Immunohistochemical analyses of human IVD tissues showed immunoreactive cells of NRTN, ARTN, PSPN, GFRA2, GFRA3, and GFRA4 in both AF and NP tissues in both the early degenerate stage and the advanced degenerate stage.

#### 2.4.1. NRTN Expression

**Figure 4:**
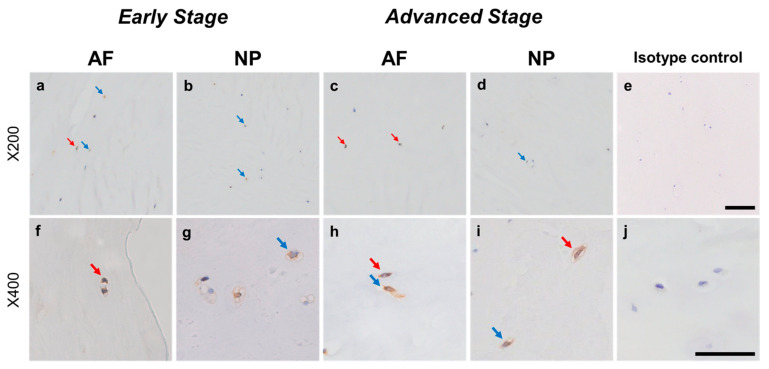
Immunohistochemical staining reveals neurturin (NRTN) in human intervertebral disc (IVD) cells in the anulus fibrosus (AF; **a**,**c**,**f**,**h**) and nucleus pulposus (NP; **b**,**d**,**g**,**i**) tissues at the early (**a**,**b**,**f**,**g**) and advanced (**c**,**d**,**h**,**i**) stages of degeneration. Low magnification images (×200; **a**–**e**) and high magnification images (×400; **f**–**j**) are shown. Isotype control (**e**,**j**). Blue arrows indicate 1+ immunostaining cells, whereas red arrows indicate 2+ immunostaining cells. Scale bar: 200 μm (**a**–**e**), 50 μm (**f**–**j**).

**Figure 5:**
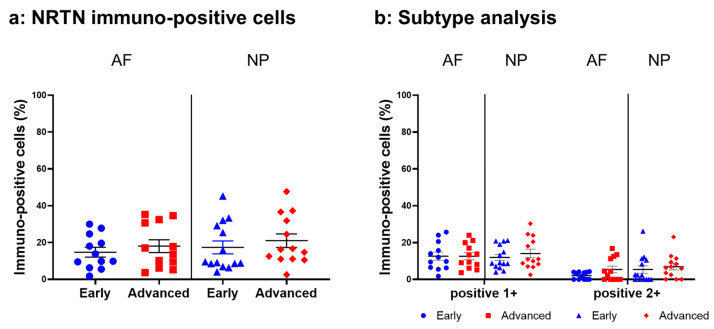
Percentage of immunopositive cells for neurturin (NRTN) in human intervertebral disc tissues from the early and advanced stages of disc degeneration (**a**) and subtype analysis by the intensity of immunostaining (**b**). AF, anulus fibrosus; NP, nucleus pulposus. Error bars: standard error (S.E.).

Relatively weak immunoreactivity against NRTN was found sparsely in cells within the AF (Figure 4a,c) and NP (Figure 4b,d) tissues, both in the early and advanced stages of degeneration. High magnification images revealed that the weak to mild immunoreactivities were identified around the nuclei of the cells in the AF (Figure 4f,h) and NP (Figure 4g,i) tissues. No immunoreactivity was found in the isotype controls (Figure 4e,j). There were no significant differences in the percentage of immunopositive cells for NRTN in both AF and NP tissues between the early stage (AF: 14.7 ± 2.7%, NP: 17.4 ± 3.5%) and advanced degenerate stage (AF: 18.0 ± 3.5%, NP: 21.0 ± 3.7%) (Figure 5a). No significant differences in the percentages of 1+ and 2+ positive cells were found between the degeneration stages (Figure 5b).

#### 2.4.2. ARTN Expression

**Figure 6:**
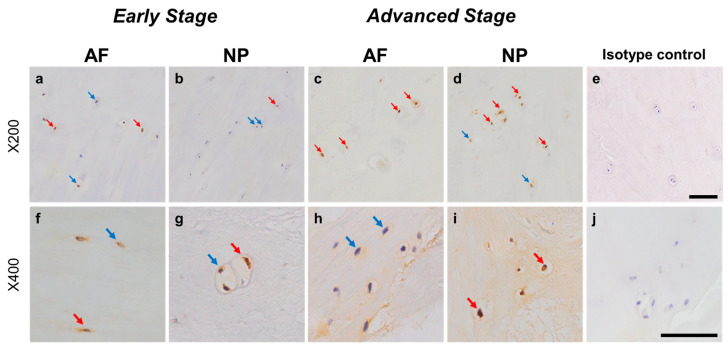
Immunohistochemical staining reveals artemin (ARTN) in human intervertebral disc (IVD) cells in the anulus fibrosus (AF; **a**,**c**,**f**,**h**) and nucleus pulposus (NP; **b**,**d**,**g**,**i**) tissues at the early (**a**,**b**,**f**,**g**) and advanced (**c**,**d**,**h**,**i**) stages of degeneration. Low magnification images (×200; **a**–**e**) and high magnification images (×400; **f**–**j**) are shown. Isotype control (**e**,**j**). Blue arrows indicate 1+ immunostaining cells, whereas red arrows indicate 2+ immunostaining cells. Scale bar: 200 μm (**a**–**e**), 50 μm (**f**–**j**).

**Figure 7:**
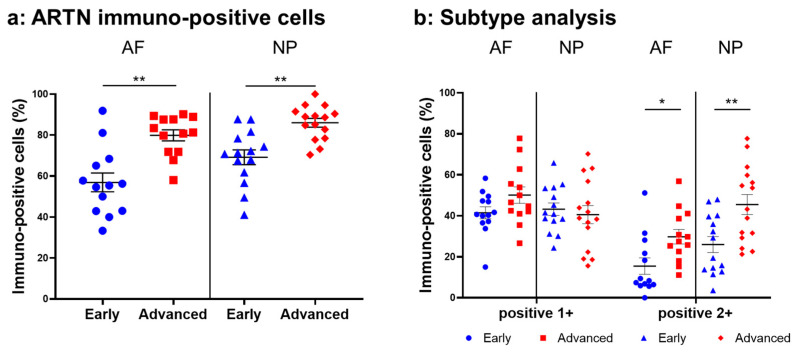
Percentage of immunopositive cells for artemin (ARTN) in human intervertebral disc tissues from the early and advanced stages of disc degeneration (**a**) and subtype analysis by the intensity of immunostaining (**b**). AF, anulus fibrosus; NP, nucleus pulposus. * *p* < 0.05 and ** *p* < 0.01 for comparisons between the early and advanced stages of degeneration by the unpaired Student’s *t* test. Error bars: standard error (S.E.).

Normal-to-intense immunoreactivity against ARTN was frequently identified in cells within AF tissues (Figure 6a,c) and NP tissues (Figure 6b,d) in the early and advanced stages of degeneration. High magnification images revealed the ARTN-immunoreactivities around the nuclei of the cells in the AF (Figure 6f,h) and NP (Figure 6g,i) tissues. No immunoreactivity was found in the isotype controls (Figure 6e,j). The percentage of ARTN-immunopositive cells in AF (79.9 ± 2.7%) and NP (86.0 ± 2.2%) tissues in the advanced degenerated stage was significantly higher than those in the early degenerated stage (AF: 56.9 ± 4.6%, *p* < 0.05; NP: 69.2 ± 3.6%, *p* < 0.01, Figure 7a). The percentages of 2+ cells of ARTN in the AF (29.8 ± 3.5%) and NP (45.5 ± 4.8%) tissues of the advanced degenerated stage were significantly higher than those in the early degenerated stage AF (15.5 ± 4.9%, *p* < 0.05) and NP (26.9 ± 3.9%, *p* < 0.01) tissues (Figure 7b).

#### 2.4.3. PSPN Expression

**Figure 8:**
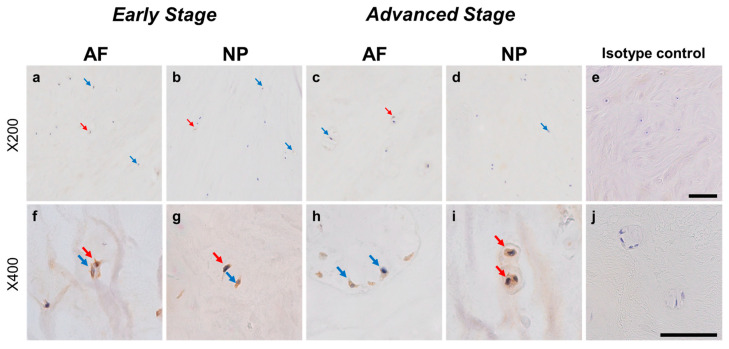
Immunohistochemical staining reveals persephin (PSPN) in human intervertebral disc (IVD) cells in the anulus fibrosus (AF; **a**,**c**,**f**,**h**) and nucleus pulposus (NP; **b**,**d**,**g**,**i**) tissues at the early (**a**,**b**,**f**,**g**) and advanced (**c**,**d**,**h**,**i**) stages of degeneration. Low-magnification images (×200; **a**–**e**) and high-magnification images (×400; **f**–**j**) are shown. Isotype control (**e**,**j**). Blue arrows indicate 1+ immunostaining cells, whereas red arrows indicate 2+ immunostaining cells. Scale bar: 200 μm (**a**–**e**), 50 μm (**f**–**j**).

**Figure 9:**
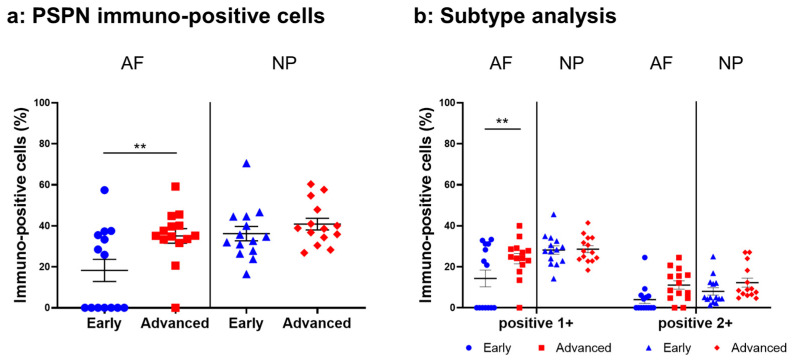
Percentage of immunopositive cells for persephin (PSPN) in human intervertebral disc tissues from the early and advanced stages of disc degeneration (**a**) and subtype analysis by the intensity of immunostaining (**b**). AF, anulus fibrosus; NP, nucleus pulposus. ** *p* < 0.01 for comparisons between the early and advanced stages of degeneration via the unpaired Student’s *t* test. Error bars: standard error (S.E.).

Immunoreactive cells against PSPN were unevenly distributed in AF (Figure 8a,c) and NP (Figure 8b,d) tissues both in early and advanced stages of degeneration. High magnification images revealed PSPN-immunoreactivities around the nuclei of the cells in the AF (Figure 8f,h) and NP (Figure 8g,i) tissues, but not in the isotype control (Figure 8e,j). The percentage of PSPN-immunopositive cells in the AF in the advanced degenerated stage (35.1 ± 3.6%) was significantly higher than that in the AF in the early degenerated stage (18.2 ± 5.4%, *p* < 0.01, Figure 9a), and the percentage of 1+ cells of PSPN in the AF in the advanced degenerated stage (24.0 ± 2.6%) was significantly higher than that in the AF in the early degenerated stage (14.3 ± 4.1% *p* < 0.01, Figure 9b). No significant differences in the percentage of PSPN-immunopositive cells in the NP were found between the early and advanced stages of degeneration (Figure 9a).

#### 2.4.4. GFRA2 Expression

**Figure 10:**
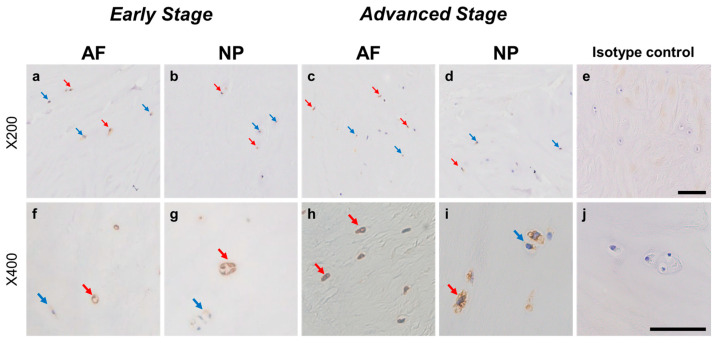
Immunohistochemical staining reveals GDNF family receptor alpha 2 (GFRA2) in human intervertebral disc (IVD) cells in the anulus fibrosus (AF; **a**,**c**,**f**,**h**) and nucleus pulposus (NP; **b**,**d**,**g**,**i**) tissues at the early (**a**,**b**,**f**,**g**) and advanced (**c**,**d**,**h**,**i**) stages of degeneration. Low-magnification images (×200; **a**–**e**) and high-magnification images (×400; **f**–**j**) are shown. Isotype control (**e**,**j**). Blue arrows indicate 1+ immunostaining cells, whereas red arrows indicate 2+ immunostaining cells. Scale bar: 200 μm (**a**–**e**), 50 μm (**f**–**j**).

**Figure 11:**
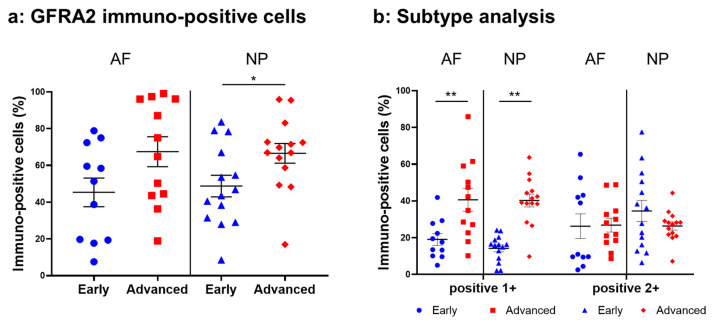
Percentage of immunopositive cells for GDNF family receptor alpha 2 (GFRA2) in human intervertebral disc tissues from the early and advanced stages of disc degeneration (**a**) and subtype analysis by the intensity of immunostaining (**b**). AF, anulus fibrosus; NP, nucleus pulposus. * *p* < 0.05 and ** *p* < 0.01 for comparisons between the early and advanced stages of degeneration via the unpaired Student’s t test. Error bars: standard error (S.E.).

Weak to intense immunoreactivity against GFRA2 was frequently found in cells within the AF (Figure 10a,c) and NP (Figure 10b,d), both in the early and advanced stages of degeneration. The immunoreactivities were mainly found in the cytoplasm of the AF (Figure 10f,h) and NP (Figure 10g,i) cells as compared to the cytoplasm of the isotype control cells (Figure 10e,j). The percentage of GFRA2-immunopositive cells in the NP in the advanced stage (67.5 ± 8.1%) was significantly higher than that in the NP in the early stage (45.3 ± 7.8%, *p* < 0.05, Figure 11a). The percentages of 1+ cells of GFRA2 in the AF and NP in the advanced stage (AF: 40.6 ± 6.2%; NP: 40.2 ± 3.5%) were significantly higher than those in the AF and NP in the early stage of degeneration (AF: 19.1 ± 3.2%; NP: 14.2 ± 1.9%, *p* < 0.01, Figure 11b).

#### 2.4.5. GFRA3 Expression

**Figure 12:**
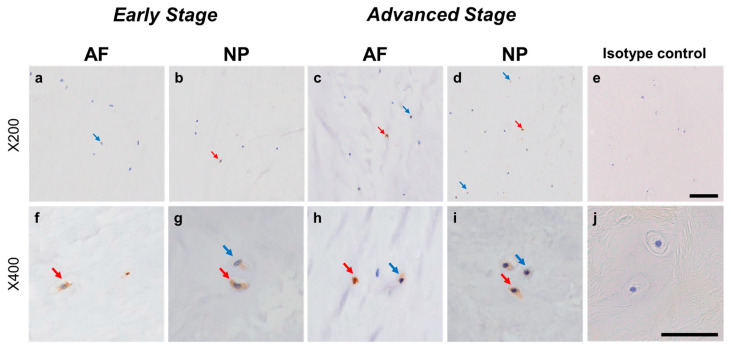
Immunohistochemical staining reveals glial-cell-line-derived neurotrophic factor family receptor alpha 3 (GFRA3) in human intervertebral disc (IVD) cells in the anulus fibrosus (AF; **a**,**c**,**f**,**h**) and nucleus pulposus (NP; **b**,**d**,**g**,**i**) tissues at the early (**a**,**b**,**f**,**g**) and advanced (**c**,**d**,**h**,**i**) stages of degeneration. Low-magnification images (×200; **a**–**e**) and high-magnification images (×400; **f**–**j**) are shown. Isotype control (**e**,**j**). Blue arrows indicate 1+ immunostaining cells, whereas red arrows indicate 2+ immunostaining cells. Scale bar: 200 μm (**a**–**e**), 50 μm (**f**–**j**).

**Figure 13:**
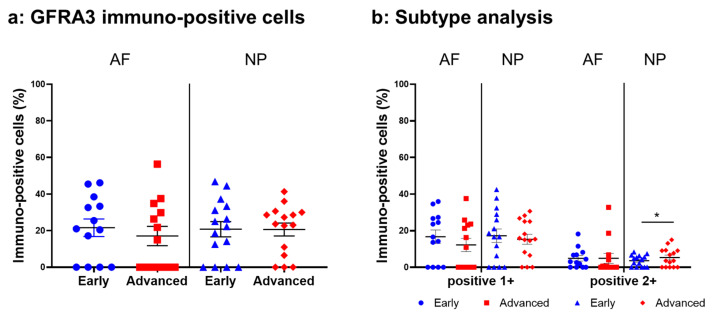
Percentage of immunopositive cells for glial-cell-line-derived neurotrophic factor family receptor alpha 3 (GFRA3) in human intervertebral disc tissues from the early and advanced stages of disc degeneration (**a**) and subtype analysis by the intensity of immunostaining (**b**). AF, anulus fibrosus; NP, nucleus pulposus. * *p* < 0.05 for comparisons between the early and advanced stages of degeneration via the unpaired Student’s *t* test. Error bars: standard error (S.E.).

Immunoreactivity against GFRA3 was sparsely identified in cells within the AF (Figure 12a,c) and NP (Figure 12b,d) both in the early and advanced stages of degeneration. The high-magnification images showed the distribution of the immunoreactivities in the cytoplasm of cells in the AF (Figure 12f,h) and NP (Figure 12g,i) tissues. No immunoreactivity was found in the isotype control (Figure 12e,j). There were no significant differences in the percentage of GFRA3-immunopositive cells in both the AF and NP between the early and advanced degenerated stages (Figure 13a). However, the percentage of 2+ cells of GFRA3 in the NP in the advanced stage (5.3 ± 1.3%) was significantly higher than that in the NP in the early stage (3.6 ± 0.8% *p* < 0.05, Figure 13b).

#### 2.4.6. GFRA4 Expression

**Figure 14:**
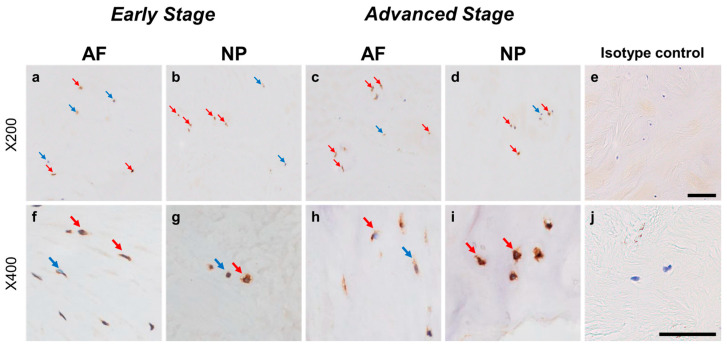
Immunohistochemical staining showing glial-cell-line-derived neurotrophic factor family receptor alpha 4 (GFRA4) in human intervertebral disc (IVD) cells in the anulus fibrosus (AF; **a**,**c**,**f**,**h**) and nucleus pulposus (NP; **b**,**d**,**g**,**i**) tissues at the early (**a**,**b**,**f**,**g**) and advanced (**c**,**d**,**h**,**i**) stages of degeneration. Low-magnification images (×200; **a**–**e**) and high-magnification images (×400; **f**–**j**) are shown. Isotype control (**e**,**j**). Blue arrows indicate 1+ immunostaining cells, whereas red arrows indicate 2+ immunostaining cells. Scale bar: 200 μm (**a**–**e**), 50 μm (**f**–**j**).

**Figure 15:**
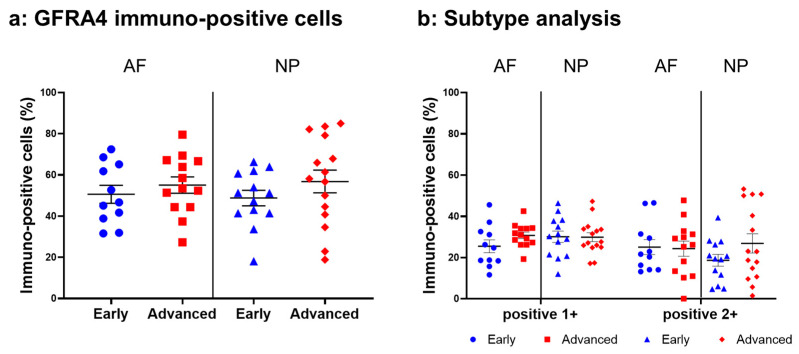
Percentage of immunopositive cells for glial-cell-line-derived neurotrophic factor family receptor alpha 4 (GFRA4) in human intervertebral disc tissues from the early and advanced stages of disc degeneration (**a**) and subtype analysis by the intensity of immunostaining (**b**). AF, anulus fibrosus; NP, nucleus pulposus. Error bars: standard error (S.E.).

Relatively intense immunoreactivity against GFRA4 was often detected in cells within the AF (Figure 14a,c) and NP (Figure 14b,d) tissues in the early and advanced stages of degeneration. High magnification images revealed GFRA4-immunoreactivities mainly in the cytoplasm of the cells in the AF (Figure 14f,h) and NP (Figure 14g,i) tissues. No immunoreactivity was found in the isotype control (Figure 14e,j). No significant differences were observed in the percentage of GFRA4-immunopositive cells between the early stage (AF: 50.6 ± 4.4%, NP: 48.8 ± 3.8%) and the advanced stage of degeneration (AF: 55.1 ± 4.0%, NP: 56.8 ± 5.5%, Figure 15a). No significant differences in the percentages of 1+ or 2+ positive cells were found between the degeneration stages (Figure 15b).

## 3. Discussion

This study showed, for the first time, that NRTN, ARTN, and PSPN and their co-receptors (GFRA2, GFRA3, and GFRA4) were expressed in human IVD cells at both the mRNA and protein level. ARTN, PSPN, and GFRA2 tended to be upregulated by pro-inflammatory stimuli (IL-1β) in both the AF and NP cells in vitro. Corresponding to the in vitro results, the percentages of ARTN-, PSPN-, and GFRA2-immunopositive cells were significantly higher in human IVD tissues in the advanced stage of degeneration than in those in the early stage of degeneration.

Previous studies have reported that GFL expression outside the nervous system was found in several human organs and tissues [12]. GFLs have been reported to play an important role in the development of epithelial structures during embryogenesis [19] and in the regulation of hematopoietic cell differentiation [20]. Interestingly, GFLs are expressed in human peripheral blood mononuclear cells, including monocytes, B lymphocytes, and T cells, which implies that GFLs may have a role in modulating immune cell responses [12,21]. In response to inflammatory stimuli, several types of immune and epithelial cells release GFLs and express their co-receptors in several tissues, which is considered to be associated with the maintenance of epithelial tissues. In the musculoskeletal system, little is known about their expression in the articular cartilage, bone, and tendon. A single study reported by Yi et al. [22] showed that GDNF mRNA expression was detected in preosteoclasts from bone-marrow-derived mice mesenchymal stem cells (BMMSCs); they showed that GDNF had the potential to simulate the osteogenic differentiation of BMMSCs [22]. The expression of GDNF in muscle has been well investigated [23,24,25,26,27]. Previous studies have demonstrated that the expression of GDNF in the rat skeletal muscle was increased by mechanical stress or denervation. Nonetheless, none of the studies assessed the overall expression of GFLs and their related receptors in the musculoskeletal system.

Several recent studies have shown that GFL expression is regulated in the inflammatory environment by lipopolysaccharides (LPS) and inflammatory cytokines (see review in [12]). In a tumor-inflammatory environment, pancreatic cancer cells upregulate GDNF release by activated macrophages following LPS stimulation [28]. GDNF mRNA expression in primary cultures of rat macrophages, microglia, and astrocytes is upregulated by LPS stimulation [29,30]. In this study, to mimic the pro-inflammatory cytokine-rich microenvironment characterizing degenerated human IVDs [31], human AF and NP cells were stimulated with IL-1β in vitro. The mRNA expression of ARTN, PSPN, and GFRA2 showed a tendency to be upregulated by IL-1β treatment in a dose-dependent manner. Accordingly, the immunohistochemical study using human IVD tissues showed that ARTN-, PSPN-, and GFRA2-immunopositive cells were significantly more abundant in the advanced degenerated disc tissues than in those of early degenerated discs. Similarly, IL-1β has been reported to significantly enhance the expression of GDNF mRNA in human AF and NP cells, with the percentage of GDNF-immunopositive cells being higher in advanced degenerated AF and NP [18].

There is increasing evidence supporting the contribution of GFLs and GDNF-sensitive neurons to regulating inflammatory pain in peripheral tissues [12]. In 2006, Malin et al. reported that GDNF, NRTN, and ARTN activated the activity of the transient receptor potential vanilloid 1 (TRPV1) in vitro [32]. They also showed that ARTN induced thermal hyperalgesia when injected into the skin, suggesting that GFLs have the potential to regulate the sensitivity of thermal nociceptors and are implicated as an essential inductor in inflammatory hyperalgesia [32]. In 2018, Nencini et al. [16] reported that inflammatory bone pain (associated with skeletal pathology, including bone cancer, osteomyelitis, osteoarthritis, and fracture) was associated with the activation and sensitization of peptidergic neurons through the ARTN/GFRA3 signaling pathway and non-peptidergic neurons through the GDNF/GFRA1 and NRTN/GFRA2 signaling pathways. They suggested that GFL signaling pathways were involved in the pathogenesis of bone pain.

Recently, Minnema et al. [33] reported that the GFRA3 and TRAPV1 receptors were upregulated in the dorsal root ganglion of the monoiodoacetate (MIA)-induced knee osteoarthritis (OA) dog model. They found that the serum ARTN concentration was significantly elevated in this animal model compared to that of a healthy dog. More recently, the same group showed that the systemic administration of the anti-ARTN antibody attenuated mechanical hypersensitivity and restored limb use in an MIA-induced knee OA mouse model [34], suggesting that ARTN/GFRA3 signaling is associated with chronic knee OA pain. In normal human IVDs, the nociceptive nerve endings (e.g., sinvertebral nerves) are distributed in the outer layer of the posterior AF [8,9]. However, along with IVD degeneration, IVD ruptures, especially AF fissures, promote the ingrowth of sensory nerve fibers immunohistochemically labeled with the protein gene product 9.5 (PGP 9.5), (calcitonin gene-related peptide) CGRP, neurofilament, or substance P toward the inner AF and NP lesions [35,36,37,38]. Using human IVD tissues, our study revealed that more than 50% of cells in AF and NP tissues express GDNF [18], ARTN, and NRTN, and that this expression increased with the progression of IVD degeneration. Therefore, we speculated that the enhanced expression of GFLs within degenerated human IVDs may sensitize sensory neurons that innervate corresponding IVDs and contribute to the pathogenesis of discogenic LBP.

In non-neural tissues, the function of GFLs in several types of epithelial cells has also been evaluated (see review in [12]). GFLs participate in protecting the intestinal epithelium by inhibiting the expression of pro-inflammatory cytokines [39]. In the human corneal epithelium, GDNF enhances cell proliferation and survival [40] and inhibits the expression of inflammatory cytokines in vitro [41]. These previous reports suggest that GFLs play a role in the process of tissue repair against injury or infection by modulating the inflammatory environment.

**Figure 16:**
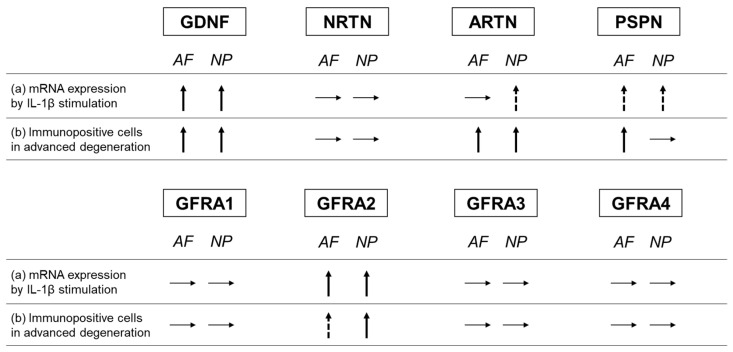
Summary of changes in the expression of GFLs and their co-receptors. (a) Changes in the mRNA expression of GFLs and their co-receptors through IL-1β stimulation. (b) Comparison of the changes in the percentage of immunopositive cells between the advanced and early stages of degeneration. Glial-cell-line-derived neurotrophic factor (GDNF), neurturin (NRTN), artemin (ARTN), persephin (PSPN), GDNF family receptor (GFR) alpha 1 (GFRA1), GFR alpha 2 (GFRA2), GFR alpha 3 (GFRA3), and GFR alpha 4 (GFRA4). Upward arrow indicates a significant increase, and dotted upward arrow indicates a trend of increase. Horizontal arrow indicates no change.

In the current study, the percentages of ARTN- and PSPN-expressing cells increased in the advanced stage of disc degeneration; however, the percentages of cells expressing their cognate co-receptors (GFRA3 and GFRA4, respectively) did not increase (Figure 16). Conversely, the percentage of NRTN-expressing cells did not increase, but that of GFRA2-expressing cells did (Figure 16). Kokaia et al. [42] evaluated the mRNA expression of GDNF and NRTN and of their co-receptors (GFRA1 and GFRA2, respectively) in a model of rat brain insults; consistent with the results of this study, they reported that the changes in the mRNA expression of the ligands and the receptors are region- and cell-specific and occur independently of each other. This suggests that the changes in the expression of GFLs and their co-receptors may occur independently of each other.

Because the expression of GFLs and their co-receptors vary independently in relation to the extent of disc degeneration, we speculate that GFLs act independently on nociceptive neurons and intervertebral disc cells. GFLs may be involved in the pain sensitivity associated with discogenic LBP, while playing a role in mediating cell metabolism and inflammatory responses in IVD degeneration.

This study had several limitations. First, human IVD tissues were obtained from spinal surgeries; these tissues were not healthy samples, but degenerated samples with different grades of degeneration. Therefore, the extent of tissue degeneration may affect the results of in vitro studies. Second, the effects of GFLs on cell survival and the matrix metabolism of human IVD cells have not been examined in this study; therefore, the relevance of GFLs with disc degeneration remains to be elucidated. Third, the extent of LBP was not evaluated in the patients whose histological samples were collected; hence, it remains unknown whether the histological expression of GHLs coincides with the extent of discogenic LBP. Third, since human IVDs contain diverse cell populations, including chondrocyte and fibroblast phenotypes and their progenitor phenotypes, the characterization of the GFL-positive cells using corresponding cell markers would be important for assessing their detailed distribution pattern in the process of degenerating human IVDs. Lastly, investigating the expression of GFLs and their co-receptors in the inflammatory cells and/or neuronal cells in the human disc tissues is important to understand the function of GFLs in the pathogenesis of discogenic LBP, which should be evaluated in future study.

## 4. Materials and Methods

### 4.1. Human IVD Tissue and Cell Isolation

This study was approved by the Institutional Committee for the Ethics of Human Research and performed in accordance with the Declaration of Helsinki. Human IVDs were obtained with informed consent from surgical specimens of spinal surgeries. For the cell culture, seven samples were obtained from seven women with lumbar spinal stenosis (LSS) (age: 59–81 years (average: 72.4 years); magnetic resonance imaging (MRI) Pfirrmann classification [43]: grade III (*n* = 1) and grade IV (*n* = 6)). Furthermore, for immunohistochemical analyses, 30 samples were obtained from 14 men and 16 women (age: 38–85 years (average: 64.5 years); MRI Pfirrmann classification: grade II (*n* = 4), grade III (*n* = 11), and grade IV (*n* = 15)). Human IVD cells were primarily cultured as a monolayer after isolation from the NP and AF tissues using sequential enzyme digestion, as previously reported [44].

### 4.2. RNA Isolation

Total RNA was isolated from human IVD cells in a monolayer culture using Isogen (NipponGene, Toyama, Japan) according to the manufacturer’s instructions. Total RNA was reverse transcribed using a first-strand cDNA synthesis kit (Roche Applied Science, Mannheim, Germany) with a DNA thermal cycler (Veriti, Applied Biosystems, Foster City, CA, USA) according to the manufacturer’s protocol.

### 4.3. Quantitative Real-Time Polymerase Chain Reaction (PCR)

The expression levels of NRTN (Hs00177922_m1, TaqMan Gene Expression Assay, Applied Biosystems), ARTN (Hs00754699_s1), PSPN (Hs03986122_s1), GFRA2 (Hs00176393_m1), GFRA3 (Hs00181751_m1), and GFRA4 (Hs00942561_g1) were quantified using real-time PCR using TaqMan Gene Expression Assay (Applied Biosystems) primer pairs for genomic TaqMan genomic assays. The assays were calibrated using 18S ribosomal RNA (Hs99999901 s1) as an internal control. To determine the expression of NRTN, ARTN, PSPN, GFRA2, GFRA3, and GFRA4, the resulting cDNA (three replicates) was amplified for the target genes. The cycle used 15 s denaturation at 95 °C and 1 min annealing and extension at 60 °C, utilizing the ABI PRISM 7000 Sequence Detection System (Applied Biosystems). The relative expression of NRTN, ARTN, PSPN, GFRA2, GFRA3, and GFRA4 was calculated using the comparative threshold method [45].

### 4.4. Effect of Interleukin-1β on Gene Expression

To examine the effects of pro-inflammatory cytokines on NRTN, ARTN, PSPN, GFRA2, GFRA3, and GFRA4, human IVD cells isolated from AF and NP tissues were cultured in the presence of interleukin-1β (IL-1β) (0.1, 1.0, and 10 ng/mL) for 48 h after serum starvation. The mRNA expression of GFLs and its co-receptors was quantified as described above.

### 4.5. Western Blotting

Cell lysates (containing 20 μg protein) from monolayer-cultured NP and AF cells were analyzed using Western blotting under reducing conditions, as previously reported [46]. The primary antibodies (1:500) were ARTN (ab178434, Abcam, Cambridge, UK), PSPN (ab198256, Abcam), GFRA2 (sc-7136, Santa Cruz Biotechnology, Dallas, TX, USA), GFRA3 (AF670, R&D systems, Minneapolis, MN, USA), and GFRA4 (MAB1439, R&D systems). β-actin served as a loading control.

### 4.6. Immunohistochemistry of Human IVD Tissues

The human IVDs obtained from spinal surgeries were divided into two groups: early-stage degeneration (MRI Pfirrmann classification: grades II and III, *n* = 15 (9 men, 6 women, 38–69 years old, average age: 55.4 years old, grades II: *n* = 4, and grade III: *n* = 11)) and advanced-stage degeneration (MRI Pfirrmann classification: grades IV, *n* = 15 (5 men, 10 women, 60–85 years old, average age: 73.7 years old)), according to the extent of disc degeneration evaluated using MRI. The samples were embedded in paraffin and serial 5 μm cross-sections were processed and stained with safranin-O/hematoxylin and eosin (safranin-O/HE) for immunohistochemical analysis. After blocking endogenous peroxidase activity, sections for NRTN, PSPN, GFRA3, and GFRA4 were heated with 0.01 M citrated buffer (pH 6.0) and those for ARTN and GFRA2 were treated with 0.04% proteinase K (pH 7.2–7.4). The sections were then incubated overnight at room temperature with the same primary antibodies used in Western blotting. The appropriate rabbit IgG (Dako), mouse IgG, or goat IgG was used as the isotype control. The sections were incubated with the appropriate horseradish peroxidase (HRP)-conjugated secondary antibody (Dako). Peroxidase activity was detected with 3, 3-diaminobenzidine tetrahydrochloride (DAB; Dojindo, Kumamoto, Japan). The sections were counterstained with Mayer hematoxylin. Immunopositive cells were classified as slightly positive (1+) or strongly positive (2+) according to the area and intensity of staining, as previously reported [47]. Positive immunoreactivities in the human IVD tissues were confirmed by comparing them with the corresponding isotype control samples. The percentages of 1+ and 2+ positive staining cells were separately quantified for each antibody by determining the mean percentage of immunopositive cells from five microscopic image fields per sample at  200× magnification.

### 4.7. Statistical Analysis

Data are expressed as the mean ± standard error. A one-way analysis of variance was used to assess the effects of culture conditions. Post hoc analyses were performed using Fisher’s least significant difference (LSD). For the histological evaluation of the expression of each protein, statistical differences between the early degenerated and advanced degenerated groups were determined using the unpaired Student’s t test. All statistical analyses were performed using IBM SPSS (IBM Japan, Tokyo). Significance was accepted at *p* < 0.05.

## 5. Conclusions

We show, for the first time, that GFLs (NRTN, ARTN, PSPN) and their respective co-receptors were expressed in human IVD cells at both mRNA and protein levels. The expression of ARTN, PSPN, and GFRA2 tended to be upregulated following IL-1β stimulation in vitro, and, furthermore, the expression was enhanced in a pro-inflammatory cytokine-rich microenvironment within degenerated human IVDs. As GFLs contribute to inflammatory pain in peripheral tissues, their expression in degenerated human IVDs may be involved in the pathogenesis of discogenic pain. Therefore, targeting GFLs could be useful when developing essential therapeutics for chronic low back pain.

## Data Availability

The data presented in this study are available upon reasonable request from the corresponding authors.
